# Chitinase 1: a novel therapeutic target in metabolic dysfunction-associated steatohepatitis

**DOI:** 10.3389/fimmu.2024.1444100

**Published:** 2024-09-23

**Authors:** Jung Hoon Cha, Na Ri Park, Sung Woo Cho, Heechul Nam, Hyun Yang, Eun Sun Jung, Jeong Won Jang, Jong Young Choi, Seung Kew Yoon, Pil Soo Sung, Si Hyun Bae

**Affiliations:** ^1^ The Catholic University Liver Research Center, Department of Biomedicine & Health Sciences, College of Medicine, The Catholic University of Korea, Seoul, Republic of Korea; ^2^ Division of Gastroenterology and Hepatology, Department of Internal Medicine, College of Medicine, Uijeongbu St. Mary’s Hospital, The Catholic University of Korea, Uijeongbu, Gyeonggi-do, Republic of Korea; ^3^ Divison of Gastroenterology and Hepatology, Department of Internal Medicine, College of Medicine, Eunpyeong St. Mary’s Hospital, The Catholic University of Korea, Seoul, Republic of Korea; ^4^ Department of Hospital Pathology, College of Medicine, Eunpyeong St. Mary’s Hospital, The Catholic University of Korea, Seoul, Republic of Korea; ^5^ Division of Gastroenterology and Hepatology, Department of Internal Medicine, College of Medicine, Seoul St. Mary’s Hospital, The Catholic University of Korea, Seoul, Republic of Korea

**Keywords:** chitinase 1, mononuclear phagocyte, metabolic dysfunction-associated steatohepatitis, inflammation, MASH mouse mod

## Abstract

**Background:**

Metabolic dysfunction-associated steatohepatitis (MASH) is characterized by persistent inflammatory cascades, with macrophage activation playing a pivotal role. Chitinase 1 (CHIT1), produced by activated macrophages, is a key player in this cascade. In this study, we aimed to explore the role of CHIT1 in MASH with progressive liver fibrosis.

**Methods:**

Fibrotic liver tissue and serum from distinct patient groups were analyzed using nCounter MAX, flow cytometry, immunohistochemistry, and enzyme-linked immunosorbent assay. A MASH mouse model was constructed to evaluate the effectiveness of OATD-01, a chitinase inhibitor. Macrophage profiling was performed using single-nuclei RNA sequencing and flow cytometry.

**Results:**

CHIT1 expression in fibrotic liver tissues was significantly correlated with the extent of liver fibrosis, macrophages, and inflammation. Single-nuclei RNA sequencing demonstrated a notable increase in macrophages numbers, particularly of lipid-associated macrophages, in MASH mice. Treatment with OATD-01 reduced non-alcoholic fatty liver disease activity score and Sirius red-positive area. Additionally, OATD-01-treated mice had lower CHIT1, F4/80, and α-smooth muscle actin positivity, as well as significantly lower levels of inflammatory markers, pro-fibrotic genes, and matrix remodeling-related mRNAs than vehicle-treated mice. Although the population of F4/80^+^CD11b^+^ intrahepatic mononuclear phagocytes remained unchanged, their infiltration and activation (CHIT1^+^MerTK^+^) significantly decreased in OATD-01-treated mice, compared with that observed in vehicle-treated mice.

**Conclusions:**

Our study underscores the pivotal role of CHIT1 in MASH. The observed significant improvement in inflammation and hepatic fibrosis, particularly at higher doses of the CHIT1 inhibitor, strongly suggests the potential of CHIT1 as a therapeutic target in MASH accompanied by progressive liver fibrosis.

## 1 Introduction

Metabolic dysfunction-associated steatohepatitis (MASH) is characterized by necroinflammation resulting from fat infiltration into hepatocytes and is accompanied by chronic liver inflammation and low-grade systemic inflammation in most affected individuals. Patients have a markedly increased risk of developing progressive liver fibrosis compared with individuals with simple steatosis (1, 2). After extensive research (3), resmetirom has recently been approved by Food and Drug Administration, becoming the first treatment agent for MASH (4). However, significant gaps persist in our understanding of MASH, and we recognize that not all patients will respond favorably to resmetirom. A deeper comprehension of the precise pathogenesis underlying MASH could expedite the exploration and development of more effective therapeutic interventions.

Mounting evidence indicates that innate immune cells, particularly macrophages, significantly contribute to the development of MASH (5, 6). Activated macrophage levels are elevated in the liver tissues of individuals with chronic liver disease, and the levels correlate with disease severity (5, 7, 8). Metabolic disorders are driven by persistent inflammatory cascades where resident hepatic macrophages (Kupffer cells, KCs) and recruited macrophages (monocyte-derived macrophages, MoMFs) play central roles (9). Additionally, liver injury and inflammation are induced by gut-derived lipopolysaccharide (LPS), activating intrahepatic immune cells, KCs, and MoMFs in patients with metabolic dysfunction-associated steatotic liver disease (MASLD) (10). Inflammatory signals increase intrahepatic mononuclear phagocyte (MP) populations, including those of KCs and MoMFs. Single-cell RNA sequencing analysis of liver specimens from amylin diet-induced MASH mice revealed enlarged MP clusters compared with those in normal mice (11). Furthermore, a recent single-cell analysis delineated distinct inflammatory phenotypes in recruited and intrahepatic MPs throughout the progression of MASH (12). This analysis was conducted on a mouse model fed a high-carbohydrate, high-fat, high-cholesterol “Western diet”, which can induce steatosis, steatohepatitis, and fibrosis, along with macrophage infiltration (12). Inflammatory MPs then activate hepatic stellate cells (HSCs), leading to collagen synthesis and consequently liver fibrosis and cirrhosis (13). Therefore, MPs, which participate in inflammatory responses and interact with hepatocytes and HSCs, emerge as promising therapeutic targets in MASH. However, the molecular mechanisms of MPs remain incompletely understood.

Chitinase 1 (CHIT1), the first genuine chitinase discovered in humans, is a chitinolytic enzyme predominantly synthesized by activated macrophages (14, 15). CHIT1 is a biochemical marker of macrophage activation and also plays a pivotal role in the inflammatory cascade (16). We previously identified a novel immune-related gene signature, including *CHIT1*, which predicts advanced fibrosis in individuals with chronic liver disease (17). Additionally, the stimulation of human monocytes/macrophages with LPS increased *CHIT1* mRNA and enzymatic activity (16). The primary objective of this study was to examine the association between elevated CHIT1 levels and disease progression in patients with liver fibrosis and MASH with progressive liver fibrosis. Additionally, we assessed the potential therapeutic role of CHIT1 in MASH by modulating macrophage activity and mitigating inflammation using mouse models of MASH-related liver fibrosis.

## 2 Materials and methods

### 2.1 Human samples

Frozen liver tissue, paraffin-embedded liver tissue sections, and serum samples were obtained from distinct groups of patients with liver fibrosis. This study included non-tumor liver tissues from 94 patients who underwent surgical resection for hepatocellular carcinoma. We successfully obtained a diverse cohort of patients representing various stages of fibrosis and inflammation by analyzing the non-tumor regions in MASH-HCC cases. Benign fibrotic liver tissues were procured from the non-tumor regions of resected livers affected by hepatocellular carcinoma. To avoid contamination by tumor cells or tumor-associated stromal cells, sufficient margins were guaranteed for non-tumor tissues. The cohort comprised patients with fibrosis stages 0 (n = 17), 1 (n = 12), 2 (n = 12), 3 (n = 25), and 4 (n = 28). The inclusion criteria were provided in a preceding publication (17). We categorized fibrosis into low-grade (F0-2, n = 41) and high-grade (F3-4, n = 53) according to a recent report demonstrating significantly worse liver-related outcomes in patients with F3 and F4 liver fibrosis (18). Frozen tissues for flow cytometry analysis were collected from patients with distinct liver fibrosis (F0-2, n = 3; F3-4, n = 3) at Seoul St. Mary’s Hospital, Catholic University of Korea (**Supplementary Table S1**). To evaluate the potential involvement of CHIT1 in MASH with progressive liver fibrosis, immunohistochemistry (IHC) was conducted on paraffin-embedded sections of liver biopsies, and enzyme-linked immunosorbent assay (ELISA) was performed on serum samples. Paraffin-embedded sections of liver biopsies from 39 patients previously diagnosed with MASH were collected at the Eunpyeong St. Mary’s Hospital of the Catholic University of Korea. The cohort included patients with fibrosis stages 0 (n = 5), 1 (n = 8), 2 (n = 7), 3 (n = 10), and 4 (n = 9) (**Supplementary Table S2**). Additionally, serum samples from individuals with liver fibrosis, comprising 62 patients with diverse liver diseases, were collected at Seoul St. Mary’s Hospital and Eunpyeong St. Mary’s Hospital of The Catholic University of Korea (**Supplementary Table S3**). The study protocol conforms to the ethical guidelines of the 1975 Declaration of Helsinki. The study was approved by the Institutional Review Boards of Ajou Medical Center (AJIRB BMR-KSP-18-444) and The Catholic University of Korea (XC20EEDI0034 and PC23SISI0123). All participants included in the study provided written informed consent. The fibrosis stage of liver tissues was assessed using the METAVIR scoring system (19, 20).

### 2.2 MASH-related liver fibrosis mouse models

We established a mouse model of MASH-related liver fibrosis by administering streptozocin (STZ, 0.2 mg; Sigma-Aldrich, St. Louis, MO, USA) in combination with a high-fat and high-cholesterol (HFHC) diet (Western Diet; D12079B, 41 kcal% fat, 43 kcal% carbohydrates; Research Diets, New Brunswick, NJ, USA) (21). This model was employed to evaluate the effects of the CHIT1 inhibitor, OATD-01, on MASH. The resultant liver pathology, encompassing steatosis, inflammation, and fibrosis, closely mirrored the progression observed in humans, ranging from fatty liver to more advanced stages, such as MASH and fibrosis. Sixteen-day-pregnant female C57BL/6 J mice were procured from the Jackson Laboratory (Japan, Tsukuba). Three-day-old male C57BL/6 J mice underwent subcutaneous injection of STZ to induce islet destruction, and they were given an HFHC diet starting at 4 weeks of age, which was maintained throughout the duration of the study. The male mice were randomly assigned to five groups: normal diet (ND, n = 11), STZ-injected normal carbohydrate diet (STZ-ND, n = 8) (Teklad Global 18% Protein Rodent Diet; TD 2018 C, Envigo, Indianapolis, IN, USA), STZ-injected HFHC diet (STZ-HFHC, n = 12), and STZ-injected OATD-01-treated HFHC diet (OATD-01-treated STZ-HFHC) (OATD-01, 30 mg/kg qd, n = 9 and 100 mg/kg qd, n = 11; MedChemExpress, Monmouth Junction, NJ, USA). A previous study has demonstrated that the oral administration of OATD-01 at 30 mg/kg twice daily effectively suppressed chitinolytic activity in fibrotic lungs (22). Therefore, OATD-01 was orally administered at two concentrations— 30 mg/kg once daily and 100 mg/kg —once daily, for a duration of 4 weeks starting when the mice were 6 weeks old. Body weight was monitored weekly for 6–10 weeks, whereas blood samples were collected biweekly from the orbital venous plexus for blood sugar level and blood chemistry analyses. Mice were intraperitoneally anesthetized with xylazine (10 mg/kg) and tiletamine/zolazepam (40 mg/kg), and their livers were removed. All animal procedures adhered to the Laboratory Animals Welfare Act, the Guide for the Care and Use of Laboratory Animals, and the Guidelines and Policies for Rodent Experiments provided by the Institutional Animal Care and Use Committee of the Catholic University of Korea (CUMC-2021-0264-01).

### 2.3 Flow cytometry analysis

Livers from the mice were dissociated using a MACS dissociation kit and the gentleMACS Dissociator (Miltenyi Biotec). Frozen human and mouse liver tissues were mashed through a cell strainer (FALCON, 352350) at 37°C in Dulbecco’s Phosphate Buffered Saline containing 5% fetal bovine serum. The supernatant was aspirated, followed by fluorescent staining using specific dyes (human: LIVE/DEAD, CD3, CD45, HLA-DR, CD14, and CD80; mouse: 7-AAD, CD45, CD11b, F4/80, and MerTK). Intracellular proteins were labeled, and CHIT1^+^ cells subjected to stimulation were subsequently fixed and permeabilized using the Fixation/Permeabilization working solution (eBioscience™, San Diego, CA, USA) for intracellular protein staining. The antibodies used are detailed in the **Supplementary Table 4.**

Recent reports indicate that MerTK is upregulated in the livers of patients with MASLD and fibrogenic rat models (23). Moreover, scRNA sequencing of human livers with cirrhosis revealed predominant expression of *MerTK* in liver macrophages (24). Our prior study validated a significant increase in MerTK^+^ mean fluorescence intensity and percentage within intrahepatic MPs in a mouse model of MASH (21). Therefore, we also verified the expression of MerTK along with CHIT1 in intrahepatic MPs. After digesting the mouse livers, we conducted flow cytometry to analyze the intrahepatic MPs of STZ-HFHC mice treated with either vehicle or OATD-01.

### 2.4 ELISA

To detect serum human chitotriosidase (CHIT1), we employed the Human CHIT1 SimpleStep ELISA^®^ Kit (abcam, #ab246541) following the manufacturer’s guidelines. Briefly, standard and 50 µl of diluted samples were applied to individual microplate wells for subsequent analysis. Subsequently, 50 µl of the antibody cocktail was added to each well, and the mixture was incubated at room temperature for 1 h with gentle shaking. Following solution removal and washing steps, 100 µl of TMB development solution was added to each well, and the plate was incubated for 10 min. The reaction was halted using the stop solution, and absorbance was promptly read at 450 nm.

### 2.5 Nuclei isolation and single-nuclei RNA sequencing analysis

Nuclei were prepared from approximately 100 mg of frozen mouse liver tissue using the Singulator 100 system (S2 Genomics). Following dissociation, debris were eliminated through a Percoll gradient. The nuclei concentration was then evaluated using a LUNA-FL™ Automated Fluorescence Cell Counter (Logos Biosystems) and nuclei morphology was scrutinized through microscopy. Subsequently, libraries were generated using the Chromium controller in adherence to the 10× Chromium Next GEM Single Cell 3’ v3.1 protocol (CG000315). Quantification of the purified libraries was performed using quantitative real-time polymerase chain reaction (qRT-PCR) according to the qPCR Quantification Protocol Guide (KAPA), and their quality was assessed using the Agilent Technologies 4200 TapeStation (Agilent Technologies). The libraries were then sequenced on the HiSeq platform (Illumina), adhering to the read length specifications provided in the user guide.

### 2.6 Single cell gene expression analysis

Single-nuclei RNA sequencing (sNuc-Seq) was conducted using frozen liver tissue samples from one normal mouse and two mice with MASH-related liver fibrosis. Single-cell gene expression data were analyzed using Cell Ranger v7.0.1 (10x Genomics; https://support.10xgenomics.com/single-cell-gene-expression/software/pipelines/latest/what-is-cell-ranger). The raw count matrices resulting from Cell Ranger ‘aggr’ were imported into Seurat 4.3.0. Raw counts, including all genes expressed in at least three cells and all cells with a minimum of 200 detected genes, were employed in downstream analysis. To account for potential multiplets and filter out low-quality or dying cells, cells with over 5,000 genes or mitochondrial counts exceeding 5% were excluded. After filtering, a selected count value of 23,855 genes across 30,989 cells was used in subsequent steps. Gene expression values were scaled and normalized for each gene across all integrated cells. Clustering and uniform manifold approximation and projection (UMAP) analysis were performed based on statistically significant principal components. Significant top cluster markers for each cluster, compared with all remaining cells, were identified using the Wilcoxon rank-sum test (min.pct = 0.25, log fc.threshold = 0.25), with only positive results retained. Additionally, Gene-Enrichment and Functional Annotation analyses for significant gene lists from two different results were performed using the g:Profiler tool (https://biit.cs.ut.ee/gprofiler/).

### 2.7 Blood chemistry analysis

Blood samples were collected every two weeks from the orbital venous plexus, and random blood glucose levels were assessed. Serum alanine aminotransferase (ALT) and aspartate aminotransferase (AST) levels were determined using a chemistry analyzer following the manufacturer’s protocol (Vettest 8008 Chemistry Analyser, IDEXX Laboratories, Westbrook, ME, USA).

### 2.8 Histological analysis

The liver was fixed in 10% formalin for 24 h before undergoing paraffin embedding. Hematoxylin and eosin (H&E), Masson’s trichrome staining, and immunohistochemistry (IHC) were performed on paraffin-embedded sections. Paraffin-embedded block cross-sections were transferred onto salinized glass slides, followed by deparaffinization using xylene and rehydration through a series of graded alcohols.

To enable antigen retrieval for IHC, microwave heating was performed for 15 min in 0.01 M citrate buffer (pH 6.0). To quench endogenous peroxidase activity, the sections were incubated with 3% hydrogen peroxide in methanol for 30 min. For human slides, incubation involved anti-CD3 (Abcam, Cambridge, UK), anti-CD68 (clone: KP1; Dako, Carpinteria, CA, USA), and anti-CHIT1 (Invitrogen, PA5-109528) antibodies. Mouse slides were treated with anti-F4/80 (Abcam, ab111101), anti-α-SMA (Sigma-Aldrich, A2547), and anti-CHIT1 (Invitrogen, PA5-109528) antibodies. Following washing, an EnVision+ system HRP-labelled polymer (Dako) was applied at 24°C for 30 min. The slides were then treated with 3,3′-diaminobenzidine for 1 min and counterstained with hematoxylin. Morphometric analysis of liver sections stained with IHC was performed on three fields (original magnification: 200×) of liver sections from 5 to 8 liver sections per group. The analysis involved quantifying three fields—one lobular and two portal tract regions—using the ImageJ 1.8.0v software (National Institutes of Health, Bethesda, MD, USA). Additionally, the co-localization of CD68 and CHIT1 was verified with double IHC using CD68 (Dako, M0814; Magenta Red: Dako, K4001) and CHIT1 (Invitrogen, PA5-109528; DAB: Dako, K4003) antibodies.

Sirius red staining was conducted using the Picro-Sirius Red Staining Kit (Abcam, Cambridge, UK) to assess collagen deposition in liver tissues. Collagen deposition was quantified by visualizing and analyzing three randomly selected fields from each slice using ImageJ 1.8.0v software (National Institutes of Health, Bethesda, MD, USA). Morphometric analysis of Sirius red-stained liver section was performed from three fields (original magnification: 100×) of 6–10 liver sections per group.

The non-alcoholic fatty liver disease (NAFLD) activity score (NAS) is a grading measure derived from numerical scores assigned to steatosis (0–3), hepatocellular ballooning (0–2), and lobular inflammation (0–3), with a resulting range of 0–8 (21). The NAS of tissue samples from the MASH mouse model utilized in this study was assessed by a single pathologist (E.S.J).

### 2.9 Western blotting

Total protein extraction was carried out using PRO-PREP protein extraction solution (iNtRON Biotechnology, Seoul, Korea). Next, the protein samples (30 µg) were separated by sodium dodecyl sulfate–polyacrylamide gel electrophoresis and then transferred to a polyvinylidene fluoride membrane (Millipore). Subsequently, the membranes were blocked with 5% skim milk for 60 min at room temperature and then incubated overnight at 4°C with primary antibodies. Following three washes, the membranes were incubated with specific secondary antibodies for 1 h at room temperature. Protein bands were visualized using EZ-Western Lumi La (Dogen, Seoul, Korea) and imaged with the LAS-4000 (Fuji-Film, Tokyo, Japan) imaging system. Antibodies used are listed in the **Supplementary Table 4.**

### 2.10 RNA isolation and qRT-PCR

To detect mRNA expression, cDNA synthesis, and TaqMan RT-qPCR analyses were performed following previously described methods (25). Target gene mRNA levels were assessed using TaqMan gene expression assay (Applied Biosystems, Foster City, CA, USA). The assay IDs for each gene are detailed in **Supplementary Table 5**. qRT-PCR was conducted using a LightCycler 480 II system (Roche Diagnostics) with LightCycler 480 Probes Master Reaction Mix (Roche) in a total reaction volume of 20 μL.

### 2.11 Statistical analyses

Continuous data are presented as mean ± standard deviation, whereas categorical data are presented as numbers or percentages. Statistical analyses were conducted utilizing GraphPad Prism version 8.0.1 (GraphPad, Inc., San Diego, CA, USA). Continuous variables were evaluated using independent t-tests. Pearson’s correlation tests were employed to investigate relationships between the two parameters. A threshold of P < 0.05 was considered statistically significant.

## 3 Results

### 3.1 CHIT1 was upregulated in patients with liver fibrosis and MASH accompanied by progressive liver fibrosis and correlated with disease severity

**Figure 1:**
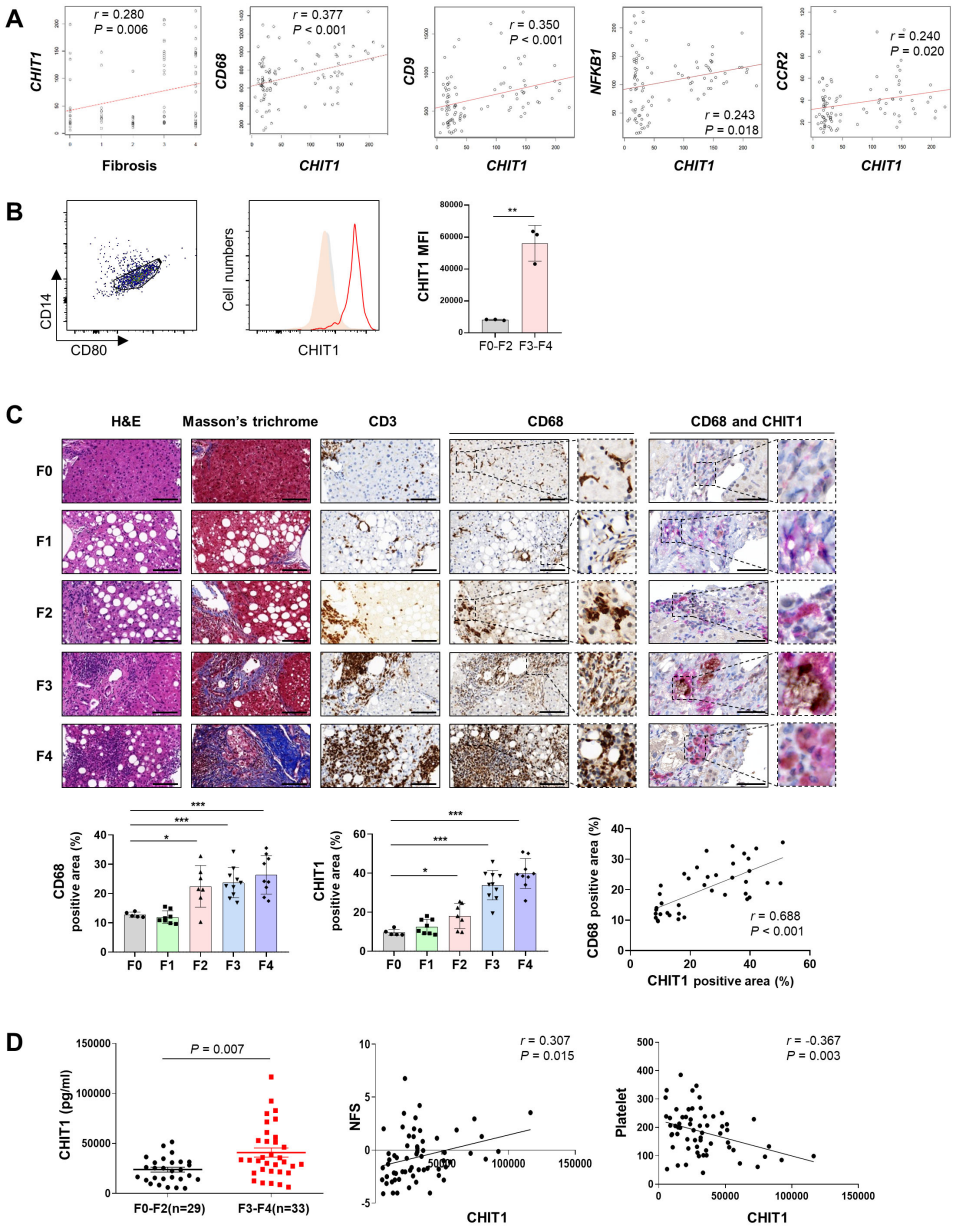
CHIT1 is upregulated in patients with liver fibrosis and MASH with progressive liver fibrosis and correlates with disease severity. **(A)** Correlation analysis of hepatic CHIT1. **(B)** Hepatic CHIT1 expression in CD14^+^CD80^+^ macrophages. **(C)** Liver biopsies stained with hematoxylin and eosin, Masson’s trichrome, CD3, CD68, and CHIT1 (scale bar: 100 µm, 400×). Double IHC for CD68 (Magenta Red) and CHIT1 (DAB Brown) (scale bar: 50 µm). Correlation analysis of hepatic CHIT1 and CD68. **(D)** Serum CHIT1 and correlation of CHIT1 with NFS and platelets. (A and C-D) Pearson’s correlation test; (B and C-D) Two-tailed independent t-test; data are presented as the mean ± SD. *P < 0.05, **P < 0.01, ***P < 0.001. CHIT1, chitinase 1; MASH, metabolic dysfunction-associated steatohepatitis; NFS, non-alcoholic fatty liver disease (NAFLD) fibrosis score; SD, standard deviation.

Correlation analysis revealed a positive association between *CHIT1* levels and liver fibrosis (r = 0.280, *P* = 0.006), as well as with the levels of the macrophage marker *CD68* (r = 0.377, *P* < 0.001) and the pro-fibrogenic macrophage marker *CD9* (r = 0.350, *P* < 0.001). The correlation between *CHIT1* levels and those of the inflammation-related genes *NFKB1* and *CCR2* was 0.243 (P = 0.018) and 0.240 (*P* = 0.020), respectively (**Figure 1A**). The expression of each gene across different stages of liver fibrosis is presented in **Supplementary Figure 1**. In patients with F3-4 liver fibrosis, there was a significant increase in the expression of *CHIT1, CD68, CD9, NFKB1*, and *CCL2* (**Supplementary Figure S1A**). We confirmed a positive correlation between *CHIT1* and *SMAD2, STAT1*, and *FN1*, genes known to be associated with liver fibrosis. The expression of these three genes also significantly increased in individuals with F3 and F4 liver fibrosis (**Supplementary Figures S1B, C**). Flow cytometry analysis of tissues from patients with distinct liver fibrosis revealed a statistically significant increase in the expression of CHIT1 in CD14^+^CD80^+^ macrophages in tissues from patients with F3-4 liver fibrosis (**Figure 1B**). Moreover, our results demonstrated that CHIT1 expression was preferentially higher in monocyte-differentiated macrophages and LPS-stimulated macrophages compared to that in human liver-derived cell lines (**Supplementary Figure S2**).

IHC analysis revealed a significant upregulation of both CD68 and CHIT1 in stage F2 or higher tissues, compared with that in F0 tissues. Moreover, a notable correlation was observed between CD68 and CHIT1 levels (r = 0.688, *P* < 0.001). Furthermore, the co-localization of CD68 and CHIT1 was confirmed using double IHC (**Figure 1C**). The ELISA results further demonstrated a significant elevation in CHIT1 levels in samples from patients with high-grade fibrosis (F3-4, n = 33), compared with that in samples from patients with low-grade fibrosis (F0-2, n = 29) (*P* = 0.007), with a notable positive correlation observed with NAFLD fibrosis score (NFS) (r = 0.307, *P* = 0.015). Additionally, a significant negative correlation with platelet count was observed (r = 0.367, *P* = 0.003) (**Figure 1D**). Overall, these results indicate a significant association between CHIT1 levels and macrophage activation, inflammation, liver injury, disease onset, and the progression to advanced liver fibrosis.

### 3.2 Intrahepatic MP population increased in mice with MASH-related liver fibrosis

**Figure 2:**
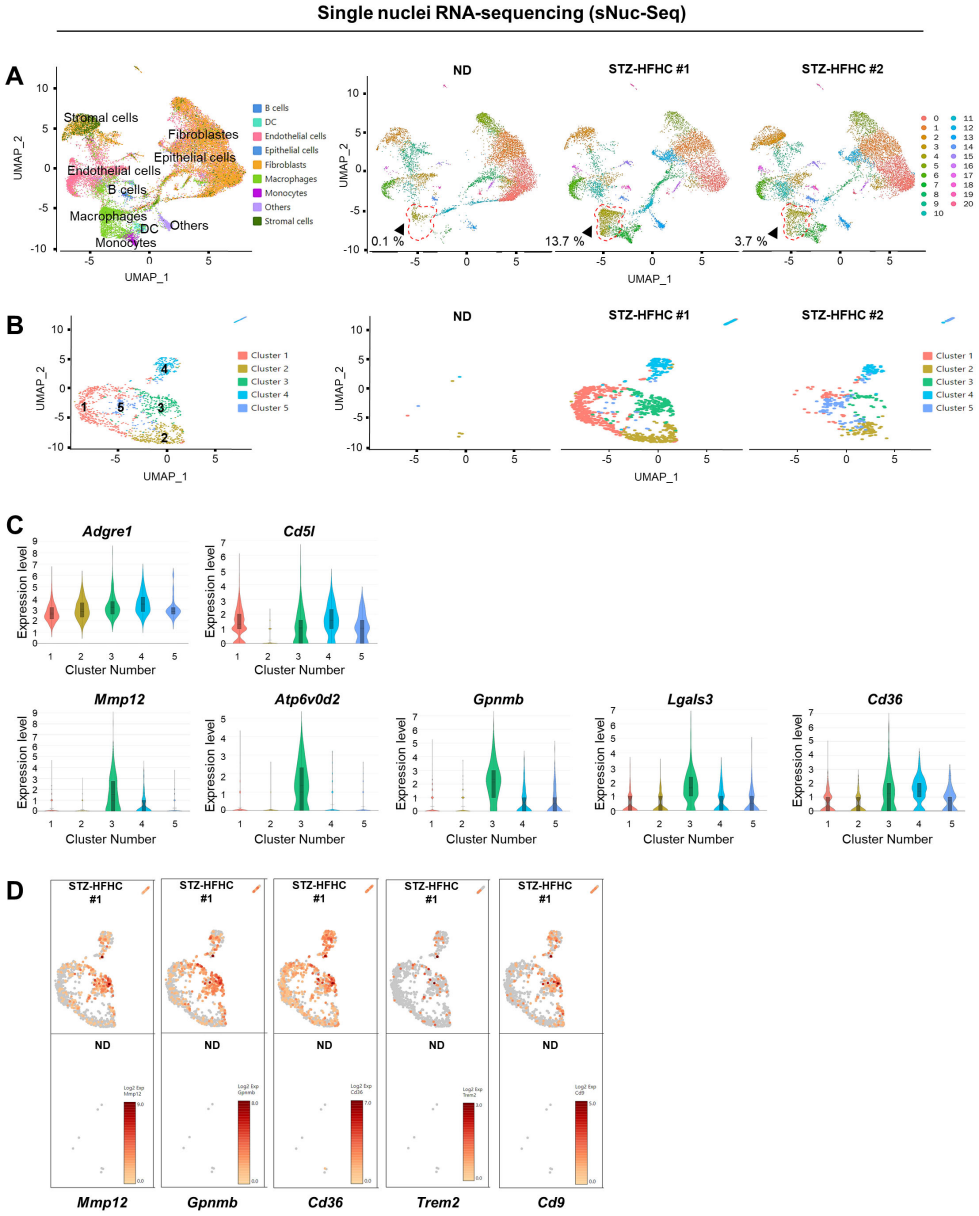
Single-nuclei RNA sequencing (sNuc-Seq) analysis of mice with MASH-related liver fibrosis. **(A)** Annotation of cell populations and uniform manifold approximation and projection (UMAP) clustering of integrated sNuc-Seq dataset. **(B)** UMAP plots of the five different macrophage subpopulations. **(C)** Violin plot of the expression of specific genes across various macrophage subpopulations. **(D)** UMAP plots of the genes associated with the lipid-associated macrophage (LAM) subtype (Mmp12, Gpnmb, and Cd36) in cluster 3 lineage. MASH, metabolic dysfunction-associated steatohepatitis.

Analysis of single-nuclei RNA sequencing (sNuc-Seq) confirmed increased intrahepatic MPs in mice (STZ-HFHC) with MASH-related liver fibrosis, compared with that in control mouse (ND). We validated the increase in macrophage numbers using the singleR package (26) (**Figure 2A**) and confirmed the rise in KCs through ScType (27) (CellMarker database; http://biocc.hrbmu.edu.cn/CellMarker/ and PanglaoDB ; https://panglaodb.se ) (**Supplementary Figure S3A**). Following the analysis of macrophage nuclei, five discrete clusters were identified, all exhibiting high *Adgre1* and *Cd5l* (**Figures 2B, C**). Interestingly, we observed increased expression of *Mmp12, Atp6v0d2, Gpnmb, Lgals3, and Cd36*, collectively known as lipid-associated macrophage (LAM) markers (28, 29), within macrophage cluster 3 (**Figure 2C**). Moreover, sNuc-Seq analysis validated the heightened expression of LAM markers, including Trem2 and Cd9 (**Figure 2D**). The analysis of KC nuclei revealed the presence of four distinct clusters, with cluster 4 exclusively identified in the normal mouse. Additionally, our findings demonstrated a close correlation with those obtained from the analysis of macrophage nuclei (**Supplementary Figures S3B–D**). These sNuc-Seq results show elevated intrahepatic MP levels in mice with MASH-related liver fibrosis, consistent with the findings from single-cell RNA sequencing analysis of MASH mouse livers (11).

### 3.3 CHIT1 inhibition mitigated liver injury in a MASH-related liver fibrosis mouse model

**Figure 3:**
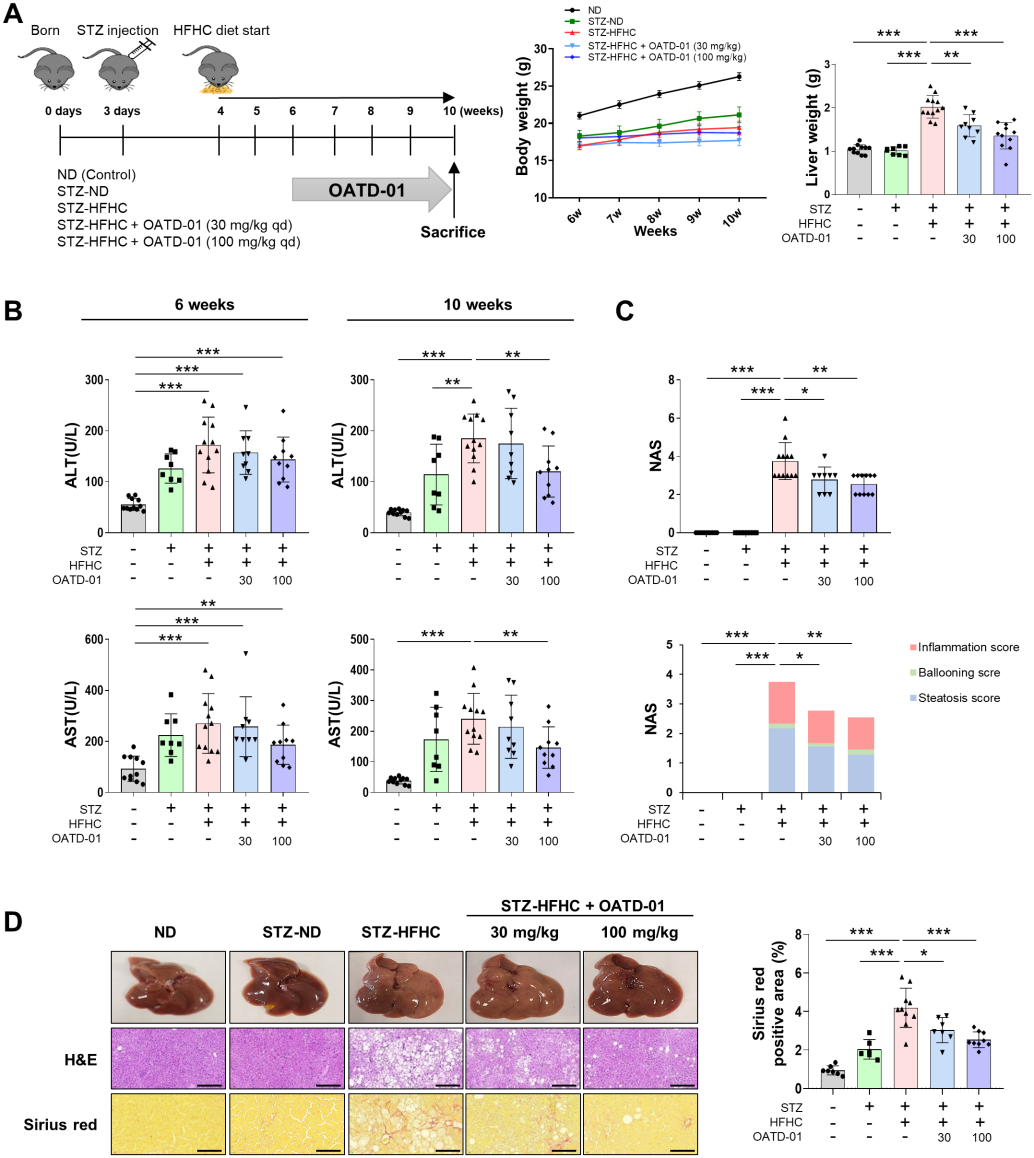
CHIT1 inhibition ameliorates liver injury in mice with MASH-related liver fibrosis. **(A)** Development of the MASH mouse model using streptozotocin and a high-fat, high-cholesterol diet. **(B)** Serum alanine aminotransferase and aspartate aminotransferase levels at 6 and 10 weeks. **(C)** NAS from mouse liver specimens (n = 8–12 per group). **(D)** Hematoxylin and eosin (scale bar: 200 µm, 200×) and Sirius red (scale bar: 100 µm, 400×) staining of liver tissues. Two-tailed unpaired independent t-test; data are presented as the mean ± SD. *P < 0.05, **P < 0.01, ***P < 0.001. CHIT1, chitinase 1; MASH, metabolic dysfunction-associated steatohepatitis; NAS, non-alcoholic fatty liver disease activity score; SD, standard deviation.

The STZ-injected group had a lower total body weight than the ND group. Conversely, liver weight significantly increased in the vehicle-treated group, while it decreased in a concentration-dependent manner in the OATD-01-treated STZ-HFHC group (**Figure 3A**). In the STZ-HFHC group, the levels of fasting blood glucose were significantly elevated (**Supplementary Figure S4A**), as was *Srebf1c* mRNA expression; however *Ppara* mRNA expression was significantly reduced (**Supplementary Figure S4B**). Furthermore, fat accumulation was evident in the STZ-HFHC group (**Supplementary Figure S4C**), indicating the successful establishment of the fatty liver model.

After establishing liver damage in the 6-week STZ-HFHC group, evidenced by elevated levels of ALT and AST, compared with those in the ND group, OATD-01 was administered for 4 weeks. Both ALT and AST levels were significantly reduced in the STZ-HFHC group treated with 100 mg/kg of OATD-01, compared with those in the vehicle-treated group (**Figure 3B**). The NAS was significantly elevated in the livers of the vehicle-treated group, whereas it decreased in a concentration-dependent manner in the livers of the OATD-01-treated STZ-HFHC group (**Figure 3C**). H&E staining of paraffin-embedded liver sections revealed a significant reduction in hepatocyte necrosis and sinusoidal congestion in the livers of the OATD-01-treated STZ-HFHC group, compared with those in the vehicle-treated group. Additionally, Sirius red staining demonstrated a concentration-dependent decrease in liver collagen deposition area in the OATD-01-treated STZ-HFHC group compared with that in the vehicle-treated group (**Figure 3D**). These findings suggest that Chit1 inhibition mitigates liver injury in a mouse model of MASH-related liver fibrosis.

### 3.4 CHIT1 inhibition attenuated hepatic inflammation and fibrosis

**Figure 4:**
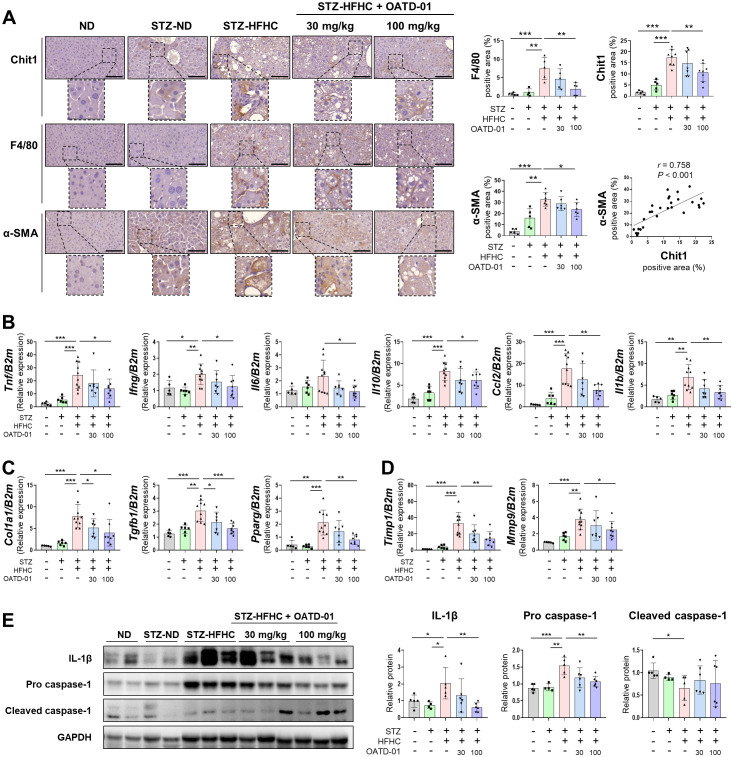
CHIT1 inhibition attenuates hepatic inflammation and fibrosis in mice with MASH-related liver fibrosis. **(A)** CHIT1, F4/80, and α-SMA immunohistochemistry (scale bar: 100 µm, 400×). Correlation analysis of hepatic CHIT1 and α-SMA (Pearson’s correlation test). **(B-D)** Hepatic mRNA levels of genes related to inflammation, fibrosis, and matrix remodeling. **(E)** Expression of proteins involved in inflammation. **(A-E)** Two-tailed unpaired independent t-test; data are presented as the mean ± SD. *P < 0.05, **P < 0.01, ***P < 0.001. CHIT1, chitinase 1; MASH, metabolic dysfunction-associated steatohepatitis; α-SMA, alpha smooth muscle actin; β2M, beta 2 microglobulin; SD, standard deviation.

IHC results revealed higher proportions of CHIT1-, F4/80-, and α-SMA-positive cells in the STZ-HFHC group than those in the ND and STZ-ND groups (**Figure 4A**). These findings are similar to those in patients with liver fibrosis and MASH accompanied by progressive liver fibrosis (**Figures 1A, C**). In the STZ-HFHC group treated with 100 mg/kg of OATD-01, the abundance of CHIT1-, F4/80-, and α-SMA-positive cells was significantly decreased, compared with that in the vehicle-treated group. IHC also confirmed a positive correlation between α-SMA and CHIT1 (r = 0.758, *P* < 0.001) (**Figure 4A**).

To further explore the molecular mechanisms underlying the anti-inflammatory and anti-fibrotic effects of OATD-01, we assessed the expression of various fibrosis markers in the liver using qRT-PCR. In the vehicle-treated group, inflammation-related genes *Tnf, Ifng, Il6, Il10, Ccl2*, and *Il1b* were markedly upregulated, whereas these genes were significantly downregulated in the OATD-01-treated STZ-HFHC group (**Figure 4B**). A similar trend was observed for fibrosis-related genes *Col1a1*, transforming growth factor beta 1 (*Tgfb1*), and *Pparg* (**Figure 4C**), as well as matrix remodeling-related genes *Timp1* and *Mmp9* (**Figure 4D)**. We observed a significant increase in the expression of IL-1β and pro-caspase-1 in the vehicle-treated group, and this was reversed by OATD-01 treatment. Conversely, cleaved caspase-1 was significantly decreased in the vehicle-treated group and increased in the OATD-01-treated STZ-HFHC group (**Figure 4E**).

THP-1 cells were differentiated into macrophages using phorbol 12-myristate 13-acetate (PMA), followed by OATD-01 administration prior to LPS activation. Consistent with observations in the MASH-related liver fibrosis mouse models, treatment with OATD-01 decreased the expression of inflammation-related genes induced by LPS (**Supplementary Figure S5**). These results suggest that inhibiting CHIT1 may mitigate liver inflammation and fibrosis in MASH-related liver fibrosis.

### 3.5 CHIT1 inhibition reduced CHIT1^+^MerTK^+^ intrahepatic MPs but not F4/80^+^CD11b^+^ MPs

**Figure 5:**
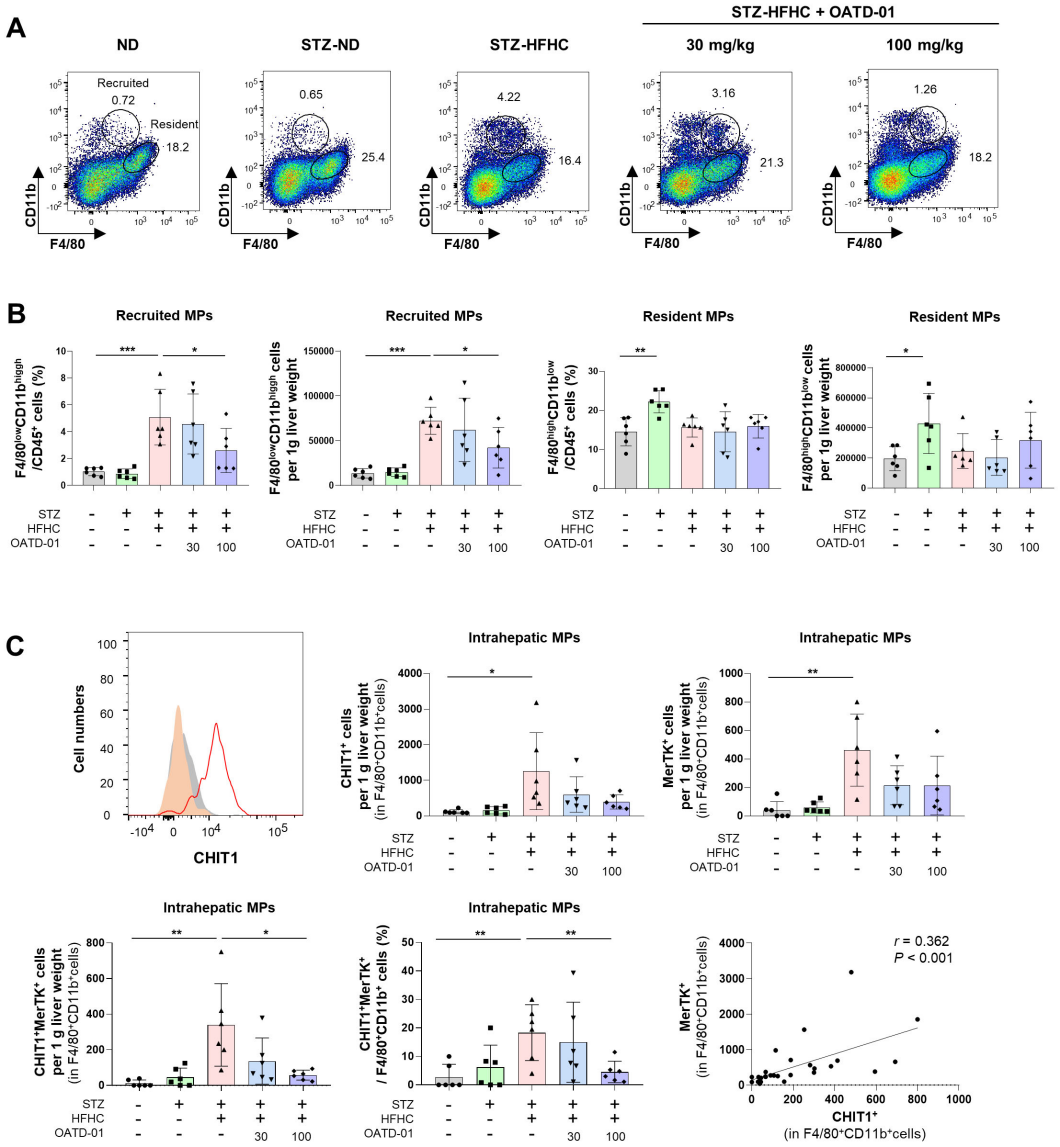
CHIT1 inhibition alters the phenotype of activated mononuclear phagocytes (MPs) in MASH. **(A)** Dot plot illustrating the proportions of recruited/resident MPs. **(B)** Recruited (F4/80^low^ CD11b^high^) and resident (F4/80^high^ CD11b^low^) MP number per g liver weight and cell percentage in vehicle or OATD-01-treated STZ-injected, HFHC diet-fed MASH mice. **(C)** CHIT1^+^, MerTK^+^, and CHIT1^+^MerTK^+^ cell number per g liver weight. Frequency of CHIT1^+^MerTK^+^ intrahepatic MP cells in STZ-injected, HFHC diet-fed MASH mice with or without OATD-01 treatment. Two-tailed unpaired independent t-test; data are presented as the mean ± SD. *P < 0.05, **P < 0.01, ***P < 0.001. Correlation analysis of hepatic CHIT1^+^ and MerTK^+^ MPs (Pearson’s correlation test). CHIT1, chitinase 1; MASH, metabolic dysfunction-associated steatohepatitis; STZ, streptozocin; HFHC, high-fat and high-cholesterol; MerTK, MER Proto-Oncogene, Tyrosine Kinase; SD, standard deviation.

**Figure 5A** illustrates the gating strategies for intrahepatic recruited (F4/80^low^ CD11b^high^) and resident (F4/80^high^ CD11b^low^) MPs. Compared with the ND group, intrahepatic (F4/80^+^CD11b^+^) MPs in the vehicle-treated group increased but did not significantly change after OATD-01 treatment. The population of resident (F4/80^high^ CD11b^low^) MPs also did not significantly differ. However, the OATD-01-treated STZ-HFHC group exhibited a significantly reduced population of intrahepatic recruited (F4/80^low^ CD11b^high^) MPs, compared with the vehicle-treated group (**Figure 5B**). After OATD-01 treatment, the number of CHIT1^+^ or MerTK^+^ intrahepatic MPs decreased. Particularly, both the number and the percentage of CHIT1^+^MerTK^+^ intrahepatic MPs significantly increased in the vehicle-treated group, while they decreased significantly in the OATD-01-treated STZ-HFHC group. Additionally, a significant correlation was observed between CHIT1^+^ and MerTK^+^ cells (r = 0.362, *P* <no><</no> 0.001) (**Figure 5C**). These results indicate that CHIT1 inhibition altered the phenotype of fibrotic macrophages rather than the total number of intrahepatic MPs.

## 4 Discussion

In this study, we found a strong correlation between increased CHIT1 levels and MASH severity, encompassing factors indicative of liver inflammation and injury. We further confirmed the potential of CHIT1 as a MASH therapeutic target. Given the prevalence of chronic hepatic inflammation and low-grade systemic inflammation among most patients with MASH, we hypothesized that controlling CHIT1 produced by activated macrophages could alleviate inflammation and serve as a potential treatment strategy for MASH. In MASH-related liver fibrosis mouse models, we demonstrated that OATD-01, a CHIT1 inhibitor, could impede liver fibrosis progression by suppressing macrophage activity and inflammation. Our findings align with the results of previous research indicating the anti-inflammatory and anti-fibrotic effects of CHIT1 inhibition observed in bleomycin-induced pulmonary fibrosis, as demonstrated by genetic inactivation of CHIT1 in *Chit1-/-* mice and in studies involving OATD-01 (22).

CHIT1 is primarily synthesized by innate immune cells, such as neutrophils, activated macrophages, and polymorphonuclear leukocytes (30). Activated macrophages subsequently generate proinflammatory cytokines, amplifying intrahepatic inflammation (31). Therefore, CHIT1 levels are low in healthy individuals but are markedly increased in response to diverse proinflammatory stimuli (32). A previous investigation revealed significantly elevated *CHIT1* levels in patients with MASH, compared with those in patients with simple steatosis (14). CHIT1 is implicated in promoting tissue remodeling in fibroblastic liver tissue, a process initiated by KCs, the liver-specific macrophages (14, 15). The overexpression of CHIT1 has been observed to influence HSC activation, as evidenced by a notable correlation between *CHIT1* and *α-SMA* expression in patients with MASH (15). We corroborated these findings in patients presenting with liver fibrosis and MASH, particularly those with advanced liver fibrosis. Moreover, we substantiated these outcomes in a mouse model of MASH-related liver fibrosis, where infiltrating macrophages assume a remarkable role. This suggests the potential involvement of CHIT1 in regulating the liver extracellular matrix and activating HSCs (15), thus contributing to the progression of liver fibrosis and cirrhosis (14, 15). It is conceivable that the release of CHIT1 contributes to the activation of non-parenchymal cells, particularly considering that the biological effects of CHIT1 are modulated by cytokine release (16). Therefore, we reiterate the hypothesis that controlling CHIT1 produced by activated macrophages could alleviate inflammation and serve as a potential therapeutic strategy for MASH.

Macrophages are the primary source of MerTK expression in livers with MASH fibrosis. In response to MerTK signaling, macrophages secrete TGF-β1 and are strategically located proximally to activate HSCs; therefore, they play a crucial role in the progression of liver injury from inflammation to fibrosis (33). Macrophages with high MerTK stimulate a profibrotic phenotype in HSCs through paracrine signaling, resulting in a substantial increase in cell migration, viability, proliferation, and expression of profibrotic factors (6, 34). Moreover, a significant correlation between *CHIT1 and α-SMA* expression has been demonstrated in patients with MASH, with CHIT1 overexpression reported to affect HSC activation (15). We demonstrated that in MASH fibrosis, CHIT1 was primarily expressed in activated macrophages and correlated with CD68, MerTK, and α-SMA. Furthermore, we validated that MASH mice exhibited a shift toward a fibrotic macrophage phenotype characterized by an increase in CHIT1^+^MerTK^+^ intrahepatic MP levels. The reduction in elevated CHIT1^+^MerTK^+^ intrahepatic MP levels in MASH mice following administration of OATD-01 affirmed CHIT1 as a therapeutic target for MASH.

The precise mechanism underlying the involvement of CHIT1 in diseases remains to be fully elucidated. Recent research has revealed a pivotal role of CHIT1 in modulating TGF-β signaling in lung fibrosis. CHIT1 plays a significant role in IL-13-induced alveolar fibrosis by modulating MAPK and TGFβ signaling in mice (35). Furthermore, CHIT1 enhances TGF-β signaling in fibroblasts, potentially by inducing TGF-β receptor expression, inhibiting its feedback inhibitor (SMAD7), and interacting with chaperone proteins such as TGF-β receptor-associated protein 1 and forkhead box O3. This augmentation leads to increased production of extracellular connective tissue, including collagen, and accumulation of extracellular matrix proteins, potentially contributing to alveolar destruction and tissue remodeling (22, 35). Moreover, fibroblasts serve as the primary effector cells responsible for fibrotic tissue responses, with TGF-β1 playing a crucial role in this process. CHIT1 amplifies TGF-β1-stimulated fibroblast proliferation and myofibroblast transformation (35).

The implication of CHIT1 in various disease contexts extends beyond its role in the human defense system against parasites. It has been linked to inflammatory diseases, asthma, acute and chronic inflammatory conditions, autoimmune disorders, dental diseases, neurological disorders, metabolic disorders, liver diseases, polycystic ovarian syndrome, endometriosis, and cancer. This wide-ranging involvement underscores the significance of ongoing research into drugs targeting CHIT1 (36). Several CHIT1 inhibitors are under investigation (36), including allosamidin (37), demethylallosamidin (35), HM508 (38), cyclic dipeptide chitinase inhibitors identified through structure-based exploration (39), and aromatic 2-(3-(methylcarbamoyl)guanidino)-N-arylacetamides (40). Recent investigations have explored the clinical potential of CHIT1 inhibitors in inflammatory and fibrotic diseases. Kasugamycin, a widely recognized aminoglycoside antibiotic, has emerged as a novel inhibitor of CHIT1, exhibiting potent anti-fibrotic effects on pulmonary fibrosis (41). OATD-01, a first-in-class chitinase inhibitor, demonstrates selective inhibition of chitinase activity against CHIT1 at low nanomolar levels (42). It is currently under investigation for the treatment of idiopathic pulmonary fibrosis (22) and severe asthma (43). OATD-01, a potent inhibitor of CHIT1 and AMCase, demonstrates robust pharmacokinetic properties in various animal models and is currently undergoing clinical trials (42).

This study had some limitations. First, we encountered difficulty in isolating KCs and MoMFs in MASH-related liver fibrosis mouse models. Unfortunately, this hindered our ability to elucidate the distinct effect of CHIT1 on MPs. Second, the precise involvement of CHIT1 in LAMs and inflammatory macrophages, both crucial in MASH progression, remains incompletely explored. Recently, lipid-loaded macrophages have emerged as a hallmark of several diseases, and macrophage clusters characterized by Trem2^+^ MASH related macrophages have been identified in diet-induced MASH mouse models (11) and humans (29). Our sNuc-Seq analysis confirmed an increase in *Trem2* and *Cd9* expression; however, it did not identify a single macrophage population among the five macrophage populations (**Figures 2C–D**). Although a previous study using human omental adipose tissue has identified *CTSB, CD9, CD36*, and *CHIT1* as highly significant differentially expressed genes in human TREM2-expressing LAMs, compared with those in macrophages (26), our results did not demonstrate elevated *CHIT1* levels. This observation likely reflects a cytoplasm-biased gene, considering that *TREM2, CD9*, and *CHIT1* are all more abundant in the cytoplasm than in the nucleus of liver cells (44). Thirdly, we were unable to identify additional critical mediators involved in CHIT1-associated pathways in MASH. Therefore, additional mechanistic analysis of CHIT1 in association with macrophage activation genes, including MerTK, GPNMB, and CD36, which play pivotal roles in the progression of MASH, is warranted.

## 5 Conclusions

Our investigation addressed the gap in the literature by elucidating the central modulatory role of CHIT1 in macrophage activity and inflammation. Through comprehensive analysis of tissue and serum samples from patients with liver fibrosis, including MASH, and validation in a MASH mouse model, we identified CHIT1 as a promising therapeutic target for MASH. CHIT1 inhibition may be a novel therapeutic strategy for treating MASH with progressive liver fibrosis.

## Data Availability

The datasets presented in this study can be found in online repositories. The names of the repository/repositories and accession number(s) can be found below: GSE269924 (GEO).
