# Supplementary material for: Chitinase 1: a novel therapeutic target in metabolic dysfunction-associated steatohepatitis
Source: Front Immunol. 2024 Sep 23;15:1444100. doi: 10.3389/fimmu.2024.1444100 (PMC11459552; doi:10.3389/fimmu.2024.1444100)
Supplement: Supplementary file 2 [file DataSheet2.docx]

Chitinase 1: A novel therapeutic target in metabolic dysfunction-associated steatohepatiti**s**

Jung Hoon Cha, Na Ri Park, Sung Woo Cho, Heechul Nam, HyunYang, Eun Sun Jung, Jeong Won Jang, Jong Young Choi, Seung Kew Yoon, Pil Soo Sung, Si Hyun Bae

**Supplementary contents**

Supplementary materials and methods---------------------------------------------------------------------- 2

Supplementary figures-----------------------------------------------------------------------------------------3

Supplementary tables------------------------------------------------------------------------------------------8

Supplementary references------------------------------------------------------------------------------------14

**Supplementary materials and methods**

**Gene expression analysis and statistical assessment of human liver tissue**

Gene expression profiles using nCounter MAX were developed according to previously described procedures (1). The correlation between the fibrosis stage and clinicopathological variables in liver tissue from 94 patients with liver fibrosis was evaluated using Pearson’s product-moment correlation. Further, significant distinctions between fibrosis stage 0 to 2 and fibrosis stages 3 and 4 were examined using the *t*-test.

**Cell line and cell culture**

Human hepatic stellate cells (HSCs; LX-2, MilliporeSigma, Burlington, MA, USA) and liver cell lines Huh7, SK-Hep-1, and SNU-449 were obtained from the Korean Cell Line Bank (KCLB, Seoul, Korea). Additionally, CD14^+^ monocytes derived from human peripheral blood mononuclear cells (PBMCs) and bone marrow-derived macrophages (BMDMs) from mice were utilized. LPS (1 µg/ml) was administered for 24 hours to CD14^+^ monocytes, CD14^+^ macrophage-like cells, and BMDMs, and for 4 hours to THP-1 macrophage-like cells. The THP-1 monocyte cell line was sourced from the Korean Cell Line Bank (KCLB, Seoul, Korea) and cultured at a density of 0.2–1 × 10^6^ cells/ml in RPMI 1640 (Welgene, LMO11-03) supplemented with 10% heat-inactivated fetal bovine serum (FBS, Gibco, 16000-044), 0.1% 2-mercaptoehanol (Gibco, 21985023), and 1% antibiotic-antimycotic (Gibco, 15240-062) at 37°C under 5% CO_2_. The THP-1 cells were utilized within 30 passages, following the manufacturer’s recommendations. For differentiation into macrophages, THP-1 monocytes (1 × 10^6^ cells/ml) were treated with 100 ng/ml phorbol 12-myristate 13-acetate (PMA) (Sigma-Aldrich, St. Louis, MO) in 100 mm dishes containing 6 ml RPMI 1640 medium over 24 h. Subsequently, the cells were maintained in PMA-free, serum-containing RPMI 1640 medium for an additional 24 h. Bacterial lipopolysaccharides (LPS) from *Escherichia col*i (TLR4 agonist) (Sigma, O111:B4) were dissolved in 1X phosphate-buffered saline (PBS). Prior to treating the cells with LPS (1 µg/ml for 22 h), the CHIT1 inhibitor, OATD-01 (2 µM and 5 µM concentrations), was pre-incubated at 37°C for 2 h.

**Oil red O staining**

Frozen sections embedded in Tissue-Tek optimal cutting temperature (OCT) compound (Sakura, Tokyo, Japan) were stained with Oil Red O kit (Abcam, Cambridge, UK) to visualize lipid accumulation in the liver. Images were captured using a Slide scanner (Pannoramic MIDI; 3DHISTECH Ltd, Hungary).

**Supplementary figures**

**Supplementary figure 1.**


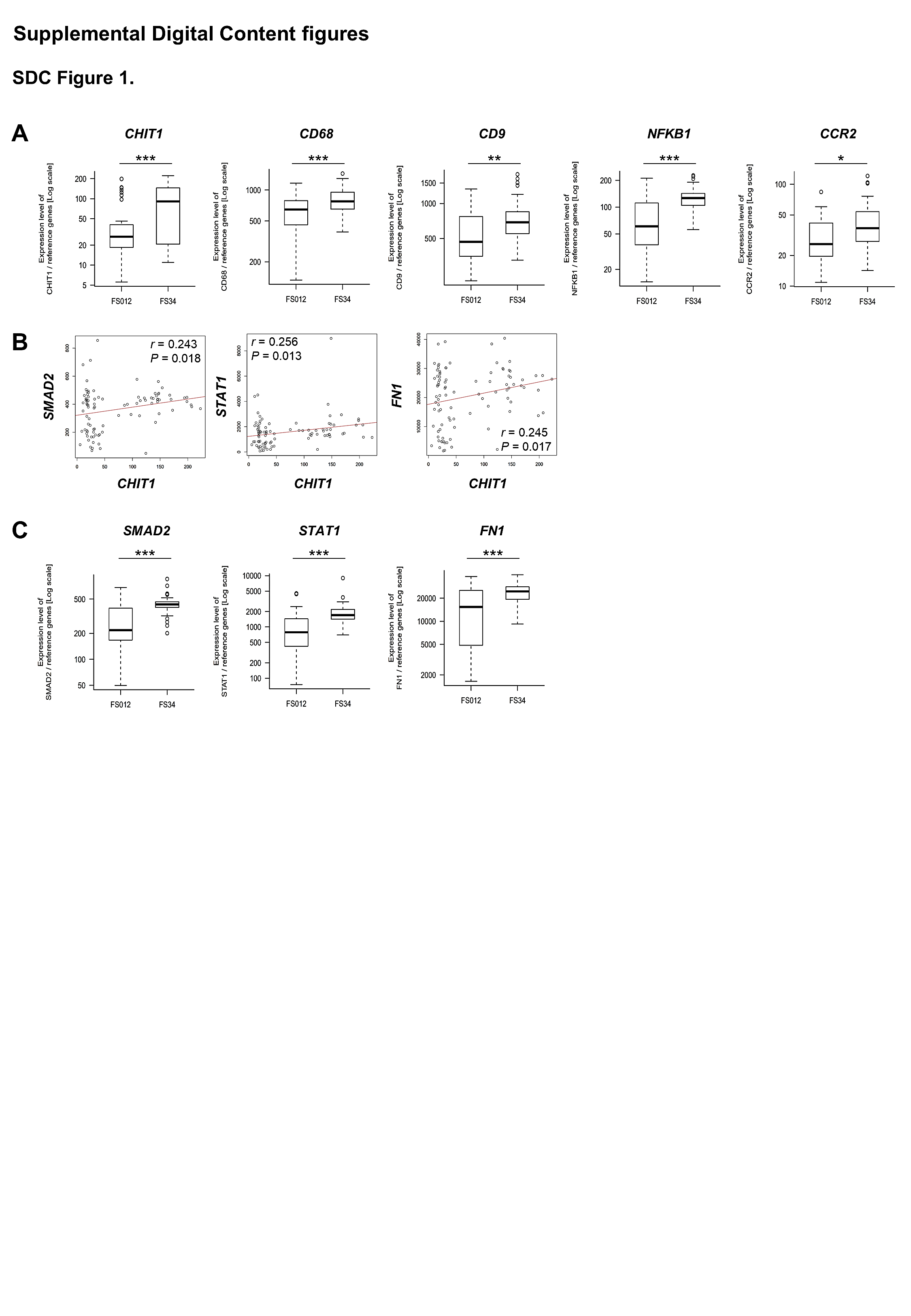


**Supplementary figure 1. Expression of each gene with liver fibrosis.** (A) Relative expressions of macrophage markers and inflammation-related genes. (B) Correlation analysis of hepatic CHTI1. (C) Relative expressions of associated genes with liver fibrosis. (A, C) Two-tailed independent t-test; (B) Pearson’s correlation test; data are presented as the mean ± standard deviation. **P* < 0.05, ***P* < 0.01, ****P* < 0.001.

FN: Fibronectin

**Supplementary figure 2.**


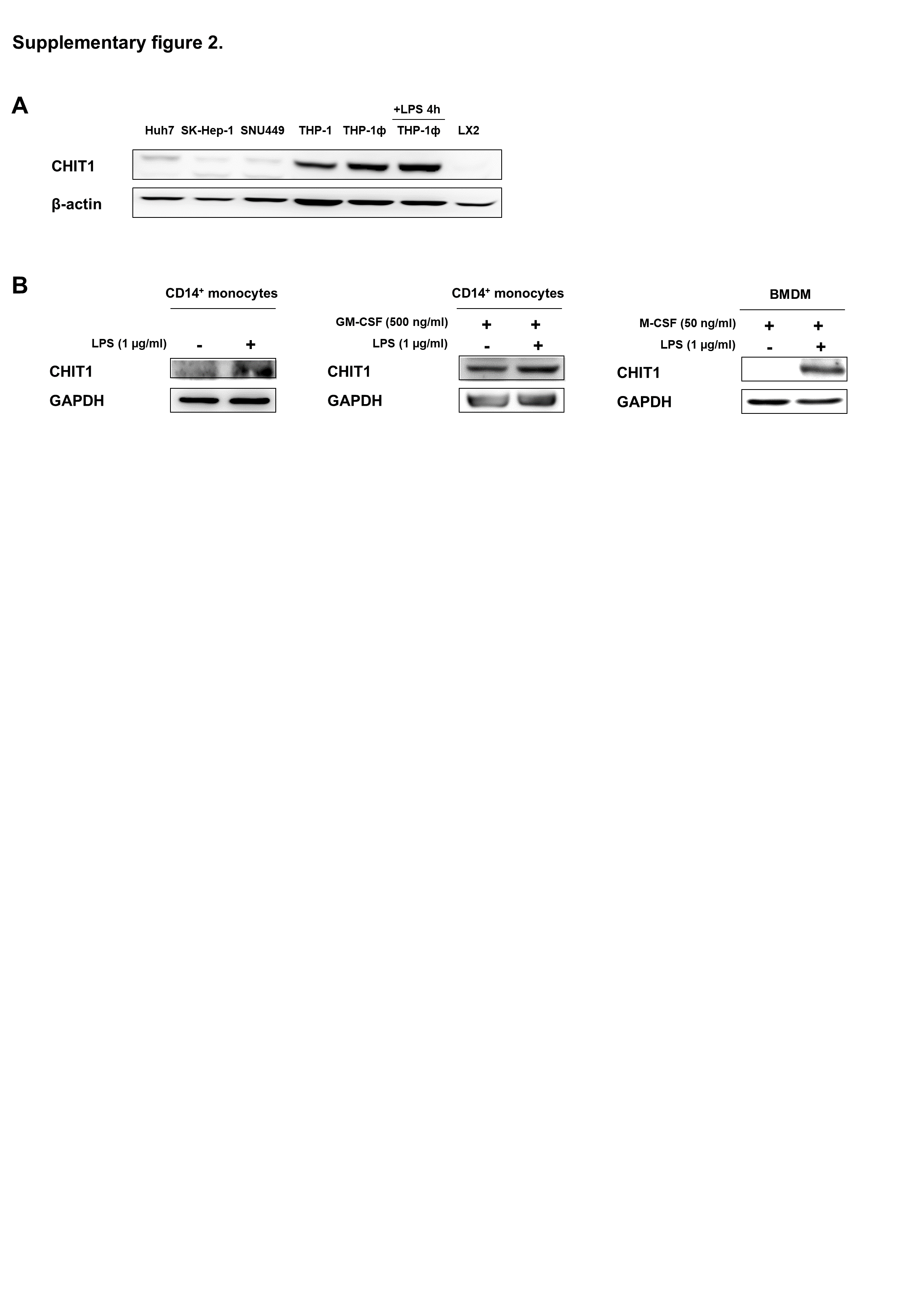


**Supplementary figure 2. Expression of CHIT1 in human cell lines.** (A) CHIT1 protein expression in human cell lines. (B) CHIT1 protein expression in LPS-stimulated CD14^+^ monocytes, CD14^+^ macrophage-like cells, and BMDMs.

CHIT1: chitinase 1; BMDM: Bone-marrow-derived macrophage

**Supplementary figure 3.**


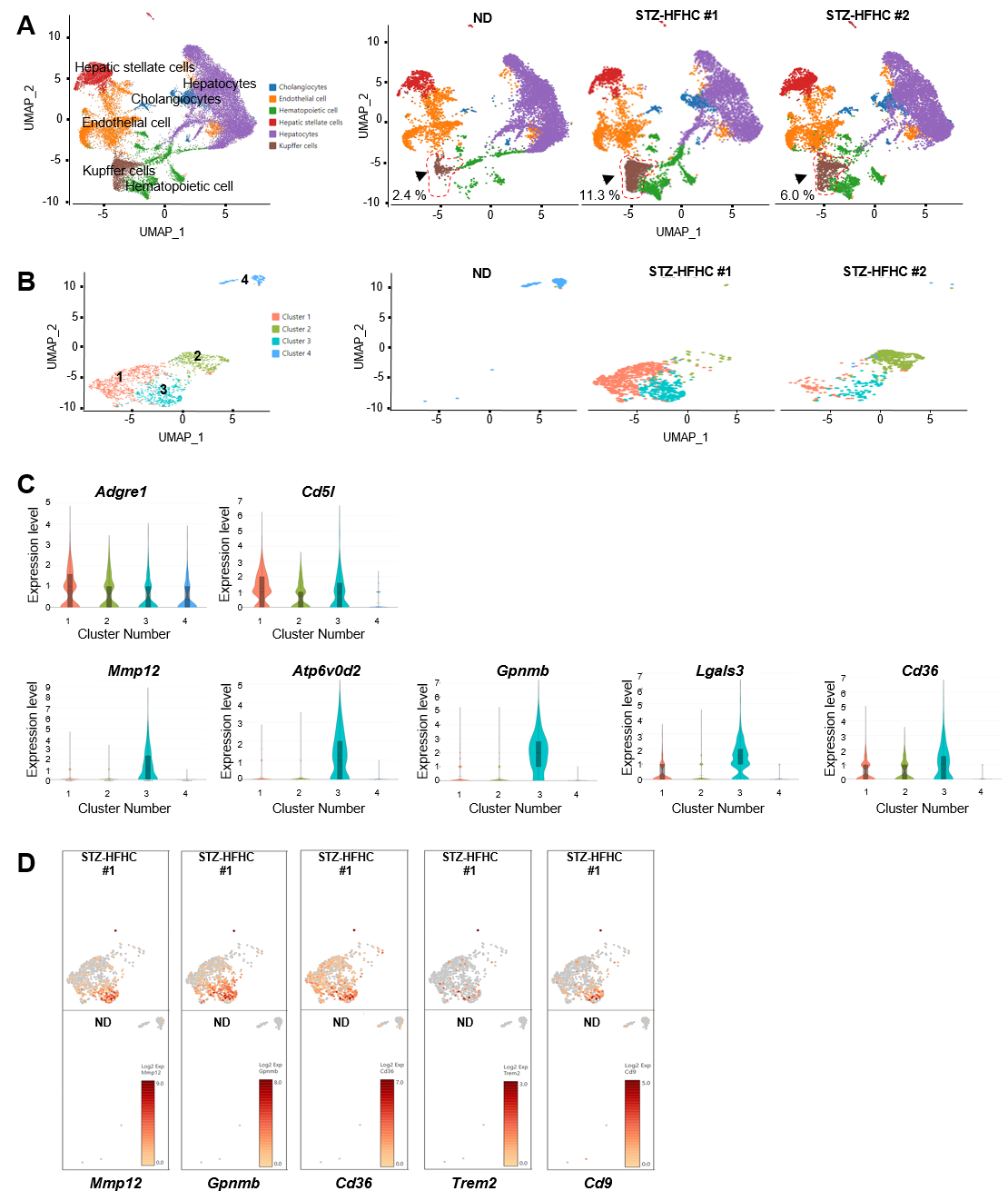


**Supplementary figure 3. Single-nuclei RNA sequencing (sNuc-Seq) analysis of mice with MASH-related liver fibrosis.** (A) Annotation of cell populations and uniform manifold approximation and projection (UMAP) clustering of integrated sNuc-Seq dataset. (B) UMAP plots of the four different kupffer cells subpopulations. (C) Violin plot of the expression of specific genes across various kupffer cells subpopulations. (D) UMAP plots of the genes associated with the lipid-associated macrophage (LAM) subtype (Mmp12, Gpnmb, and Cd36) in cluster 3 lineage.

MASH: metabolic dysfunction-associated steatohepatitis

**Supplementary figure 4.**


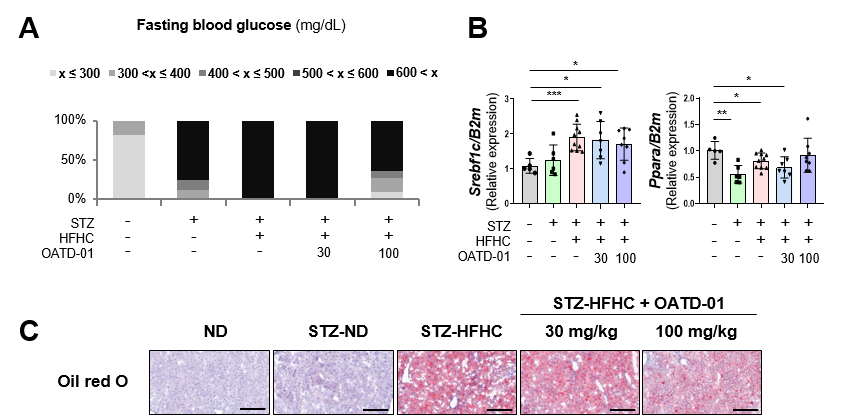


**Supplementary figure 4. Characteristics of CHIT1 inhibition mice with MASH-related liver fibrosis.** (A) The levels of fasting blood glucose at 10 weeks. (B) Hepatic mRNA levels of genes related to metabolic. Two-tailed unpaired independent t-test; data are presented as the mean ± SD. **P* < 0.05, ***P* < 0.01, ****P* < 0.001. (C) Oil red O (scale bar: 200 µm, 200×) staining of liver tissues.

CHIT1: chitinase 1; MASH: metabolic dysfunction-associated steatohepatitis; β2M: beta 2 microglobulin; SD: standard deviation

**Supplementary figure 5.**


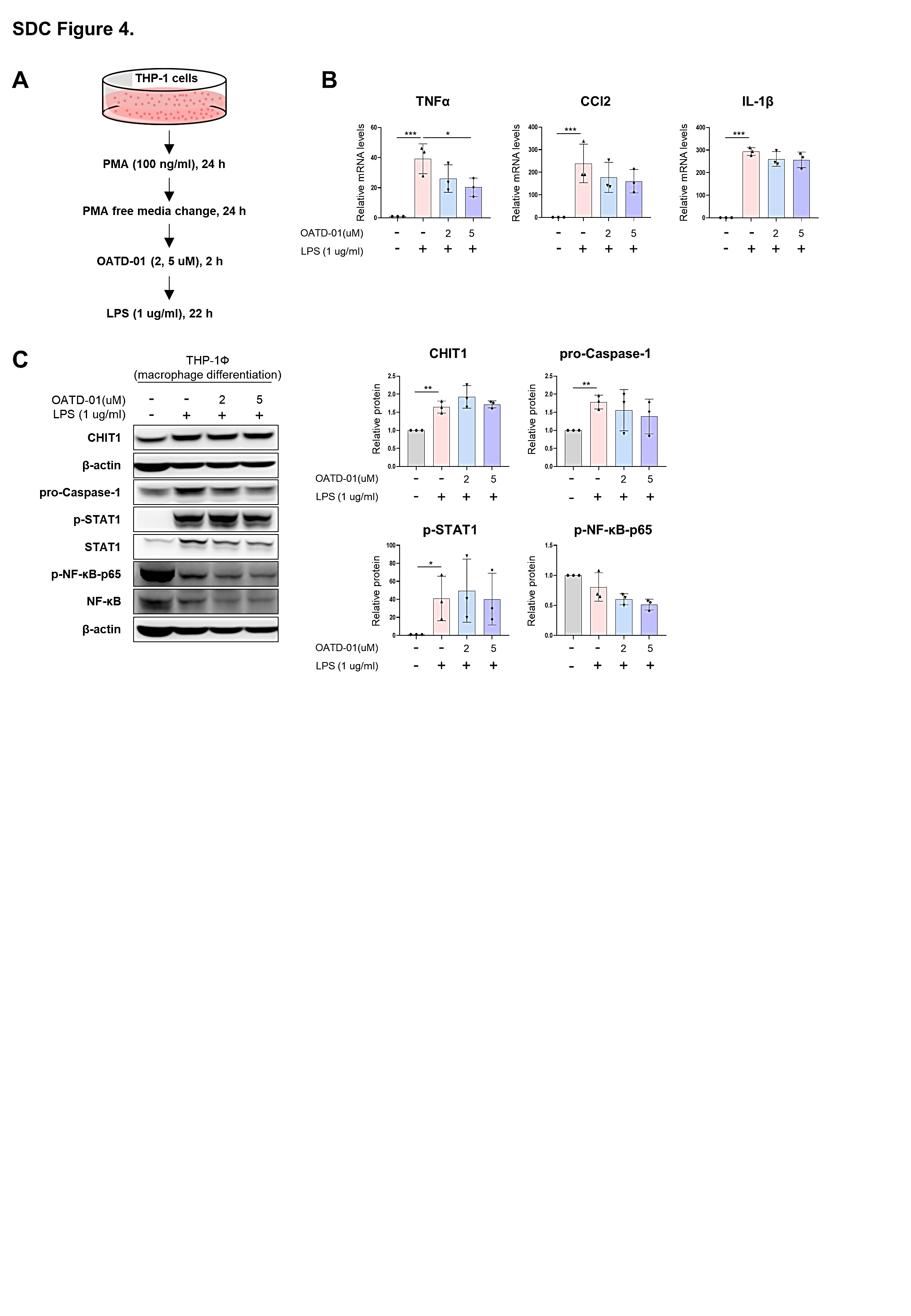


**Supplementary figure 5. Inhibition of CHIT1 mitigated inflammation in LPS-stimulated THP-1 macrophage-like cells.** (A) Experimental scheme of LPS-stimulated THP-1 macrophage-like cells. (B) mRNA levels of genes related to inflammation. (C) Expression of proteins involved in inflammation. (B, C) Two-tailed unpaired independent t-test; data are presented as the mean ± SD. **P* < 0.05, ***P* < 0.01, ****P* < 0.001.

OATD-01: chitinase 1 inhibitor; PMA: phorbol 12-myristate 13-acetate; LPS: lipopolysaccharide; SD: standard deviation

**Supplementary tables**

**Supplementary table 1**

Baseline characteristics of patients included in flow cytometry analysis.

| Characteristic | Fibrosis Stage 0-2 | Fibrosis Stage 3-4 |
| --- | --- | --- |
| Patients No. | 3 | 3 |
| Age (years) | 64.0 ± 5.2 | 66.7 ± 5.5 |
| Gender (% male) | 3 (100.0) | 3 (100.0) |
| Etiology |  |  |
| ALD |  | 1 (33.3) |
| MASH | 1 (33.3) | 2 (66.7) |
| Other | 2 (66.7) |  |
| ALT (IU/L) | 174.0 ± 101.5 | 233.0 ± 101.1 |
| AST (IU/L) | 147.0 ± 99.2 | 151.0 ± 37.5 |
| Platelets (X10^9^/liter) | 164.3 ± 12.7 | 125.3 ± 37 |
| BMI (kg/m^2^) | 22.2 ± 1.1 | 24.4 ± 2.1 |

MASH: metabolic dysfunction-associated steatohepatitis; ALD: alcoholic liver disease; ALT: alanine aminotransferase; AST: aspartate aminotransferase; BMI: body mass index.

**Supplementary table 2**

Baseline characteristics of patients included in the immunohistochemistry analysis.

| Characteristic | Fibrosis Stage 0 | Fibrosis Stage 1 | Fibrosis Stage 2 | Fibrosis Stage 3 | Fibrosis Stage 4 |
| --- | --- | --- | --- | --- | --- |
| Patients No. | 5 | 8 | 7 | 10 | 9 |
| Age  (years) | 57.2 ± 13.6 | 47.6 ± 17.6 | 49.3 ± 19.4 | 60.2 ± 15.8 | 57.7 ± 13.0 |
| Gender  (% male) | 3 (60.0) | 5 (62.5) | 1 (14.3) | 5 (50.0) | 2 (22.2) |
| BMI  (kg/m^2^) | 25.8 ± 4.8 | 28.2 ± 4.3 | 27.7 ± 4.5 | 30.1 ± 5.6 | 31.0 ± 6.1 |
| Etiology |  |  |  |  |  |
| diabetes | 1 (20.0) | 0 (0.0) | 0 (0.0) | 2 (20.0) | 0 (0.0) |
| MASH | 4 (80.0) | 8 (100.0) | 7 (100.0) | 8 (80.0) | 9 (100) |
| Cholesterol  (nmol/L) | 202.0 ± 48.6 | 186.6 ± 29.2 | 188.4 ±27.0 | 151.3 ± 24.9 | 182.1 ± 41.6 |
| Triglycerides  (nmol/L) | 143.8 ± 75.4 | 1543.3 ± 70.5 | 104.6 ± 35.0 | 145.0 ± 82.7 | 155.1 ± 81.2 |
| Glucose  (nmol/L) | 120.4 ± 23.7 | 121.0 ± 23 | 122.6 ± 39.8 | 118.4 ± 27.5 | 126.1 ± 12.6 |
| Platelets  (X10^9^/liter) | 237.4 ± 66.8 | 267.6 ±60.7 | 253.1 ± 62.3 | 216.8 ± 56.2 | 182.9 ± 62.5 |
| ALT (IU/L) | 50.2 ± 33.4 | 68.9 ± 48.8 | 169.6 ± 145.8 | 45.9 ± 35.3 | 53.8 ± 26.5 |
| AST (IU/L) | 92.4 ± 68.8 | 53.4 ± 33.0 | 102.0 ± 53.0 | 50.2 ± 25.3 | 65.9 ± 36.0 |
| GGT (IU/L) | 54.5 ± 33.6 | 48.8 ± 26.4 | 65.7 ±54.1 | 52.8 ± 28.2 | 76.0 ± 41.4 |
| Steatosis grade | 1.3 ± 0.5 | 1.6 ± 0.7 | 1.9 ± 0.7 | 1.6 ± 0.5 | 1.3 ± 0.5 |
| Inflammation  grade | 1.0 ± 0 | 1.6 ± 0.5 | 1.3 ± 0.5 | 1.6 ± 0.7 | 1.8 ± 0.8 |
| Hepatocellular  ballooning | 0.8 ± 0.5 | 0.4 ± 0.5 | 1.3 ± 1.0 | 1.0 ± 0.0 | 1.8 ± 0.8 |
| NAFLD score | 2.6 ± 1.1 | 3.6 ± 1.5 | 4.4 ± 1.4 | 2.9 ± 1.2 | 3.9 ± 2.1 |

BMI: body mass index; MASH: metabolic dysfunction-associated steatohepatitis; ALT: alanine aminotransferase; AST: aspartate aminotransferase; GGT: gamma glutamyl peptidase; NAFLD score: non-alcoholic fatty liver disease activity score.

**Supplementary table 3**

Baseline characteristics of patients included in enzyme-linked immunosorbent assay.

| Characteristic | Fibrosis Stage 0-2 | Fibrosis Stage 3-4 |
| --- | --- | --- |
| Patients No. | 29 | 33 |
| Age (years) | 50.3 ± 14.4 | 60.9 ± 11.5 |
| Gender (% male) | 10 (34.5) | 16 (48.5) |
| BMI (kg/m^2^) | 24.2 ± 4.6 | 24.9 ± 4.6 |
| Etiology |  |  |
| CHB | 13 (44.8) | 15 (45.5) |
| CHC | 6 (20.7) | 3 (9.1) |
| ALD | 1 (3.4) | 6 (18.2) |
| MASH | 1 (3.4) | 6 (18.2) |
| Other | 8 (27.6) | 3 (9.1) |
| NFS | -2.0 ± 1.4 | 0.6 ± 2.2 |
| Platelets  (X10^9^/liter) | 238.1 ± 64.9 | 136.1 ± 53.2 |
| ALT (IU/L) | 50.9 ± 38.6 | 55.5 ± 39.0 |
| AST (IU/L) | 47.9 ± 28.0 | 67.9 ± 54.5 |
| TB (mg/dl) | 0.7 ± 0.2 | 1.2 ± 0.8 |
| Fibrosis |  |  |
| stage 0 | 0 (0.0) | 0 (0.0) |
| stage 1 | 23 (79.3) | 0 (0.0) |
| stage 2 | 6 (20.7) | 0 (0.0) |
| stage 3 | 0 (0.0) | 22 (66.7) |
| stage 4 | 0 (0.0) | 11 (33.3) |

BMI: body mass index; CHB: chronic hepatitis B; CHC: chronic hepatitis C; ALD: alcoholic liver disease; MASH: metabolic dysfunction-associated steatohepatitis; NFS: non-alcoholic fatty liver disease fibrosis Score; ALT: alanine aminotransferase; AST: aspartate aminotransferase; TB: total bilirubin.

**Supplementary table 4**

Antibodies used in this study

| **Name** | **Citation** | **Supplier** | **Cat no.** | **Clone no.** |
| --- | --- | --- | --- | --- |
| LIVE/DEAD™ Fixable Violet Dead Cell Stain Kit | P. R. Roh, et al. Biomed Pharmacother. 2022 Dec:156:113952. | Invitrogen | L34955 | - |
| BD Horizon™ V450 Mouse Anti-Human CD3 | RRID:AB_1645570 | BD Biosciences | 560365 | Monoclonal (UCHT1) |
| CD45 PE-conjugated Antibody | Cong Li, et al. Stem Cell Rev Rep. 2022 Aug;18(6):2059-2073. | R&D Systems | FAB1430P-100 | Monoclonal (2D1) |
| APC-H7 Mouse Anti-Human HLA-DR | RRID:AB_10611876 | BD Biosciences | 561358 | G46-6 |
| CD14 Monoclonal Antibody (61D3), PE-eFluor™ 610, eBioscience™ | RRID:AB_2574534 | Invitrogen | 61-0149-42 | Monoclonal (61D3) |
| PE anti-human CD80 Antibody | RRID:AB_314504 | Biolegend | 305208 | Monoclonal  (2D10) |
| 7-AAD | RRID:AB_2869266 | BD Biosciences | 559925 | - |
| V500 Rat Anti-Mouse CD45 | RRID:AB_10697046 | BD Biosciences | 561487 | 30-F11 |
| V450 Rat anti-CD11b | RRID:AB_1645266 | BD Biosciences | 560456 | M1/70 |
| APC/Cyanine7 anti-mouse F4/80 Antibody | RRID:AB_893477 | Biolegend | 123118 | Monoclonal  (BM8) |
| MERTK Monoclonal Antibody (DS5MMER), PE-Cyanine7, eBioscience™ | RRID:AB_2573466 | Invitrogen | 25-5751-82 | Monoclonal (DS5MMER) |
| Goat anti-Rabbit IgG (H+L) Cross-Adsorbed Secondary Antibody, APC | RRID:AB_2534068 | Invitrogen | A-10931 | Polyclonal |
| CHIT1 Polyclonal Antibody | RRID:AB_2854939 | Invitrogen | PA5-109528 | Polyclonal |
| Chitotriosidase (A-11) | Jonathan Yap, et.al  Front Physiol. 2020 Jun 23:11:714. | Santa Cruz Bio-technology | sc-271460 | monoclonal |
| Anti-CD3 antibody | RRID:AB_306429 | Abcam | ab828 | Polyclonal |
| Anti-CD68 antibody | RRID:AB_2314148 | Agilent Dako | M081401-2 | Monoclonal  (KP1) |
| Anti-F4/80 antibody | RRID:AB_10859466 | Abcam | ab111101 | Monoclonal  (SP115) |
| Monoclonal Anti-Actin, α-Smooth Muscle | RRID:AB_476701 | Sigma-Aldrich | A2547 | Monoclonal  (1A4) |
| IL-1β (3A6) Mouse mAb | RRID: AB_2715503 | Cell Signaling Technology | 12242 | 3A6 |
| Anti-pro Caspase-1 + p10 + p12 antibody [EPR16883] | RRID:AB_2884954 | Abcam | ab179515 | EPR16883 |
| Cleaved Caspase-1 (Asp296) (E2G2I) Rabbit mAb | RRID: AB_2923067 | Cell Signaling Technology | 89332 | E2G2I |
| Phospho-Stat1 (Tyr701) (58D6) Rabbit mAb | RRID:AB_561284 | Cell Signaling Technology | 9167 | 58D6 |
| Stat1 (D1K9Y) Rabbit mAb | RRID:AB_2737027 | Cell Signaling Technology | 14994 | D1K9Y |
| Phospho-NF-κB p65 (Ser536) (93H1) Rabbit mAb | RRID: AB_331284 | Cell Signaling Technology | 3033 | 93H1 |
| NF-κB p65 (D14E12) XP® Rabbit mAb | RRID:AB_10859369 | Cell Signaling Technology | 8242 | D14E12 |
| GAPDH (14C10) Rabbit mAb | RRID: AB_561053 | Cell Signaling Technology | 2118 | 14C10 |
| Monoclonal Anti-β-Actin antibody produced in mouse | RRID:AB_476744 | Sigma-Aldrich | A5441 | Monoclonal  (AC-15) |

**Supplementary table 5**

TaqMan® Gene Expression Assays IDs used in this study

| **Name** | **Assay ID / Catalog #** | **Supplier** |
| --- | --- | --- |
| B2m (mouse) | Mm00437762_m1 / #4351370 | Thermo Fisher Scientific |
| Tnf (mouse) | Mm00443258_m1 / #4331182 | Thermo Fisher Scientific |
| Ifng (mouse) | Mm01168134_m1 / #4331182 | Thermo Fisher Scientific |
| Il-6 (mouse) | Mm00446190_m1 / #4331182 | Thermo Fisher Scientific |
| Il-10 (mouse) | Mm00439614_m1 / #4331182 | Thermo Fisher Scientific |
| Ccl2 (mouse) | Mm00441242_m1 / #4331182 | Thermo Fisher Scientific |
| Il1b (mouse) | Mm00434228_m1 / #4331182 | Thermo Fisher Scientific |
| Col1a1 (mouse) | Mm00801666_g1 / #4331182 | Thermo Fisher Scientific |
| Tgfb1 (mouse) | Mm01178820_m1 / #4331182 | Thermo Fisher Scientific |
| Pparg (mouse) | Mm00440940_m1 / #4331182 | Thermo Fisher Scientific |
| Timp1 (mouse) | Mm01341361_m1 / #4331182 | Thermo Fisher Scientific |
| Mmp9 (mouse) | Mm00442991_m1 / #4331182 | Thermo Fisher Scientific |
| Srebf1 (mouse) | Mm00550338_m1 / #4331182 | Thermo Fisher Scientific |
| Ppara (mouse) | Mm00440939_m1 / #4331182 | Thermo Fisher Scientific |
| GAPDH (human) | Hs02786624_g1 / #4351370 | Thermo Fisher Scientific |
| TNF (human) | Hs00174128_m1 / #4331182 | Thermo Fisher Scientific |
| CCL2 (human) | Hs00234140_m1 / #4331182 | Thermo Fisher Scientific |
| IL1B (human) | Hs01555410_m1 / #4331182 | Thermo Fisher Scientific |

**References**

1. Sung PS, Kim CM, Cha JH, Park JY, Yu YS, Wang HJ, et al. A Unique Immune-Related Gene Signature Represents Advanced Liver Fibrosis and Reveals Potential Therapeutic Targets. *Biomedicines* (2022) 10(1). Epub 20220116. doi: 10.3390/biomedicines10010180.
